# Supplementary material for: Diverse biophysical and molecular mechanisms drive phytoplankton sinking in response to starvation
Source: PLoS Biol. 2025 Nov 19;23(11):e3003508. doi: 10.1371/journal.pbio.3003508 (PMC12668614; doi:10.1371/journal.pbio.3003508)
Supplement: S1 Table — (PDF) [file pbio.3003508.s009.pdf]

Table S1. Molecular (dry) density values used in simulations.

Molecular species	*Density (g/ml)*	*Reference*	*Additional notes*
Protein	1.35	[1,2] (BNIDs 110540, 114284)	Generic protein values range from 1.31 to 1.37
Lipid	0.92	[1] (BNID 114321)	Combined values of triolein, trilinolein, and tricaprylin
Carbohydrate	1.5	[1] (BNIDs 103206, 112354, 112354)	Combined values of starch granules
Water	1.0		Reflects H_2O independently of any salts
Other	1.3		Estimated density of all other cellular components

1. Milo, R., Jorgensen, P., Moran, U., Weber, G. & Springer, M. BioNumbers—the database of key numbers in molecular and cell biology. *Nucleic Acids Res* **38**, D750–D753 (2010).
2. Fischer, H., Polikarpov, I. & Craievich, A. F. Average protein density is a molecular-weight-dependent function. *Protein Science* **13**, 2825 (2004).
