# Supplementary material for: Diverse biophysical and molecular mechanisms drive phytoplankton sinking in response to starvation
Source: PLoS Biol. 2025 Nov 19;23(11):e3003508. doi: 10.1371/journal.pbio.3003508 (PMC12668614; doi:10.1371/journal.pbio.3003508)
Supplement: S2 Table — The fractional (w/w) dry content values are derived from literature and the fractional water content values are derived from experiments in this paper. (PDF) [file pbio.3003508.s010.pdf]

Table S2. Cell composition values used in simulations. The fractional (w/w) dry content values are derived from literature and the fractional water content values are derived from experiments in this paper.

Species name	***Dry content***				***Water*** **(v/v total)**	***Ref.***
	Protein **(w/w dry)**	***Lipid*** **(w/w dry)**	***Carbohydrate*** **(w/w dry)**	***Other*** **(w/w dry)**		
Dunaliella tertiolecta	49%	17%	17%	18%	52.6%	[1–3]
Chaetoceros calcitrans	29%	13%	18%	40%	80.9%	[4,5]
Phaeodactylum tricornutum	45%	9%	18%	29%	67.1%	[6,7]
Generic	45%	15%	20%	20%	66.7%	

1. Kent, M., Welladsen, H. M., Mangott, A. & Li, Y. Nutritional Evaluation of Australian Microalgae as Potential Human Health Supplements. *PLoS One* 10, e0118985 (2015).
2. Fábregas, J., Patiño, M., Arredondo-Vega, B. O., Tobar, J. L. & Otero, A. Renewal rate and nutrient concentration as tools to modify productivity and biochemical composition of cyclostat cultures of the marine microalga Dunaliella tertiolecta. *Appl Microbiol Biotechnol* 44, 287–292 (1995).
3. Kobbia, I. A., Khalil, Z., Asker, M. S. & Abd-Elsayed, S. M. Effect of Nitrogen on the Biochemical Constituents and Antioxidant Production by Two Green Unicellular Algae. *Egyptian Journal of Phycology* 11, 151–170 (2010).
4. Harrison, P. J., Thompson, P. A. & Calderwood, G. S. Effects of nutrient and light limitation on the biochemical composition of phytoplankton. *J Appl Phycol* 2, 45–56 (1990).
5. de Vela Bastos, C. R. Industrial Production of Diatoms Skeletonema Costatum and Chaetoceros Calcitrans. *PQDT - Global* (Universidade do Algarve (Portugal), 2021).
6. Branco-Vieira, M. *et al.* Biochemical characterization of Phaeodactylum tricornutum for microalgae-based biorefinery. *Energy Procedia* 153, 466–470 (2018).
7. KaiXian, Q. & Borowitzka, M. A. Light and nitrogen deficiency effects on the growth and composition of Phaeodactylum tricornutum. *Appl Biochem Biotechnol* 38, 93–103 (1993).
