# Supplementary material for: Diverse biophysical and molecular mechanisms drive phytoplankton sinking in response to starvation
Source: PLoS Biol. 2025 Nov 19;23(11):e3003508. doi: 10.1371/journal.pbio.3003508 (PMC12668614; doi:10.1371/journal.pbio.3003508)
Supplement: S3 Table — (PDF) [file pbio.3003508.s011.pdf]

Table S3. Details of the phytoplankton species studied.

Species name	***CCMP identifier***	***Culture media***	***Motility***	***Additional notes***
Chlamydomonas sp.	CCMP222	L1-Si	Motile	
Dunaliella tertiolecta	CCMP362	L1-Si	Motile	
Phaeodactylum tricornutum	CCMP632	L1	Non-motile	
Prymnesium parvum	CCMP708	L1-Si	Motile	Toxic
Tetraselmis sp.	CCMP908	L1-Si	Motile	
Chaetoceros calcitrans	CCMP1315	L1	Non-motile	
Isochrysis galbana	CCMP1323	L1-Si	Motile	
Heterosigma akashiwo	CCMP1870	L1-Si	Motile	Toxic
Emiliania huxleyi	CCMP2090	L1-Si	Non-motile	Non-calcifying
