# Supplementary material for: Diverse biophysical and molecular mechanisms drive phytoplankton sinking in response to starvation
Source: PLoS Biol. 2025 Nov 19;23(11):e3003508. doi: 10.1371/journal.pbio.3003508 (PMC12668614; doi:10.1371/journal.pbio.3003508)
Supplement: Attachment — Submitted filename: Wu et al 2025_Point-by-point reviewer replies_PLOS Biology.pdf [file pbio.3003508.s015.pdf]

PLOS Biology Submission (PBIOLOGY-D-25-02800R1)

We thank the reviewers and the editor for their feedback. Below, we provide a point-by-point reply to reviewer comments. Our replies are in blue.

Reviewer #1:

Wu et al. conducted an elegant and careful study that combines biophysical modeling with cell measurements to investigate how nutrient starvation affects their sinking behavior of marine phytoplankton. They found that most species sink faster under starvation, although the underlying mechanisms vary among taxa. In some species, accelerated sinking results from higher cell density, whereas in others it reflects changes in cell volume. Notably, lipid accumulation as observed in Phaeodactylum tricornutum, decreases cell density and slows sinking velocity. Starch accumulation is also involved in the sinking velocity under starvation in green algae species. These findings demonstrate that phytoplankton employ diverse biophysical and molecular strategies to regulate buoyancy, thereby facilitating vertical migration and contributing to oceanic carbon flux under nutrient-limited conditions. The strength of this manuscript is its comparative analysis across nine marine phytoplankton species, which allows the authors to dissect mechanistic insights to biological significance in an area that remains under studied. Overall, the manuscript is clearly written and logically organized. The research motivation is compelling, and the authors present comprehensive data to support their conclusion. I strongly recommend this manuscript for publication in PLOS Biology.

We thank the reviewer for the constructive and encouraging feedback.

Minor points:

Line 79: (Chartoceros calcitrans in FigS1B or Phaeodactylum tricornutum in Fig1C)

Thank you for spotting this error, which we have now corrected.

Line 112: Remove extra "that"

Thank you for spotting this error, which we have now corrected.

Line 113: Isochrysis galbana showed no change in velocity. If measurements of dry volume, density, and lipid/protein/water content, are already available, it would be interesting to compare these with the other two haptohytes. Such comparisons could reveal whether compensatory changes among cellular components account for the lack of change in estimated sinking velocity.

Thank you for pointing out that this would be of interest. Unfortunately, we do not have such data available, but we fully agree with the reviewer's notion that compensatory changes in cell composition could exist. We will consider such investigations in our future work.

Figures S5A and S6: Consider moving some of these data to Figure 4, if there are sufficient space to include them alongside the modeled values.
We have tried the reviewer's suggestion, but this would result in the same data and/or analysis being separated into multiple figures, which may confuse the reader. We have opted to keep our figures as they are.

Fig 2D: If Y axis represents each species, why the bar shows slope. At first, I thought the slope has analytical meaning, then I realize that this is probably a presentation adjustment to avoid overlap among the bars. In any cases, an explanation would help. Alternatively, consider plotting the bars in parallel to simplify interpretation.
Thank you for pointing out this important source of confusion. We have now provided a better explanation of our data presentation in our figure legend by stating that: "Species are in the same order as in (C), separated by dotted horizontal lines. Note that paired high and low nutrient condition replicates are connected by a solid line."

Figure 3A is a summary of all the data, but it is somewhat difficult to see. You may change the background color or label contrast, which will improve readability.
Thank you for noting this. We have now increased label size, increased arrow size, and slightly changed the background color to increase figure clarity. The updated figure is shown below. We have not changed the color coding, as we want to ensure the figure is suitable for color blind individuals (we've checked this for protanopia and deuteranopia-type color blindness).

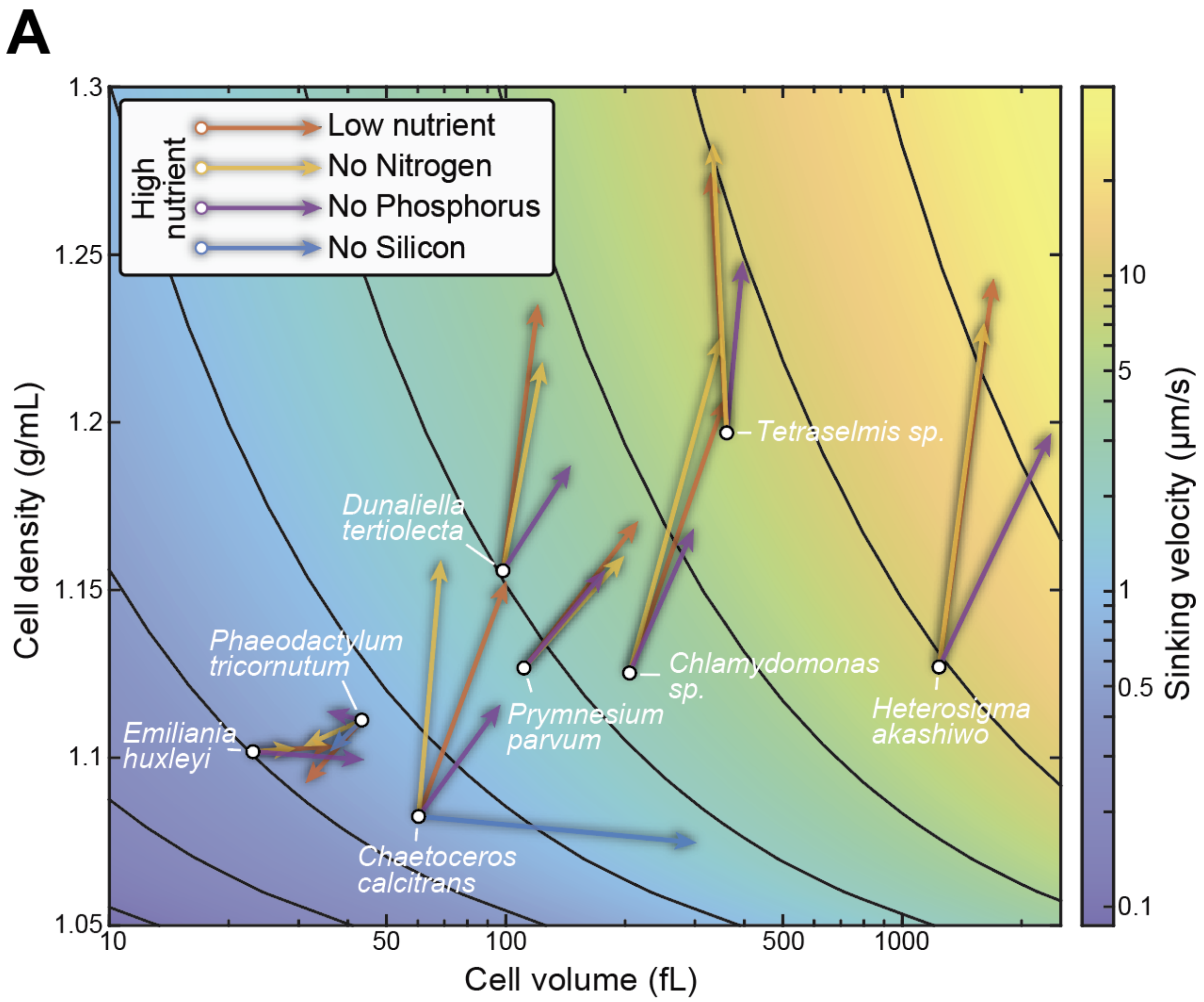

Fig S3A: Provide a little more clarity on calculation of proliferation rates and the exact meaning of the numbers in Y axis, in the methods section and/or in figure legends and graph axis titles.

We have now added the following text to the figure legend of Fig S3A: "Proliferation rates reflect the number of cell doublings / day, and these were calculated from cell counts measured on days 4, 5, and 6, following starvation start (cell mass and volume measurements were carried out on day 5)."

Fig S3B Legend: "Brightfield images were used for instead of fluorescence imaging" should be "Brightfield images were used instead of fluorescence imaging"

Thank you for spotting this error, which we have now corrected.

Fig S4 appears before Fig 3B in the text in page 6.

The reviewer is correct that we have cited Fig S4 before Fig S3B. Our supplemental figures are organized by topic and/or experimental approach. We cite each supplemental figure in order (S1, S2, S3, etc.), but some subpanels are only cited later in our main text. In this case, Fig S3A was cited on page 4 of our main text, before any citations to Fig S4.

Reviewer #2:

This paper provides a thorough quantitative investigation of sinking velocities upon starvation across 9 phytoplankton species. High quality measurements of cell volume and density using a state-of-the art technique (SMR) are combined with an interesting model which allows deciphering the respective contribution of volume increase vs. density changes in cell sinking. In the last part of the paper, the molecular changes driving cell density variations (i.e., lipid vs. starch accumulation) are investigated by combining EM imaging and drug treatments. Overall, this is a high-quality quantitative investigation; the model provides interesting insights, the experimental measurements are of high-quality and the findings raise interesting future research directions. I fully recommend this paper for publication.

We thank the reviewer for the constructive and encouraging feedback.

I only have few recommendations, mostly aimed at easing the reader's understanding.

1) The paper sometimes skips important methodology description which would make the findings more convincing and easier to understand. (i) For example, results in Figure 3 -B are really interesting (and nicely displayed) but it is not possible to understand how they were obtained from reading the main text (how the respective influence of cell volume vs. density on sinking velocity was calculated). The model is a strength of this paper and it could be better explained in the main text. (ii) Another example is that references 32-34 are important to understand how data were acquired/quantified and 1-2 sentences summarizing what these previous findings were would be helpful.

Thank you for highlighting these shortcomings of our writing. We've now added more details about our methodology to the results section. More specifically, we state on line 159 that "We then sought to decouple cell density and volume from Eq (1) using the first order Taylor approximation (see Materials and Methods) in order to determine their relative contributions to cell sinking under each starvation condition", and on line 186 that "We then compared how cellular water and dry content changes influence cell sinking using a Taylor expansion-based approach (see Materials and Methods)".

We have also clarified our methods and the references 32-34 to the reader. More specifically, we now state on line 95, where we first introduce our experimental approach to

determine cell sinking, that "In this approach, we measure single-cell volumes using Coulter counter, single-cell buoyant masses using a suspended microchannel resonator (SMR), and cell shapes using microscopy". We also now explain the basic idea of our intracellular water and dry content measurement on line 178, where we state that: "We used a previously established approach [ref 32], where cells' buoyant masses are determined in normal and heavy water to determine the dry volume of the cells (i.e., dry content), which can then be compared to the total volume of the cells to determine the water volume (i.e., water content)".

2) The discussion is interesting but perhaps a bit dense. (i) The first paragraph emphasizes the interest of the model which provides estimates for how buoyancy could be achieved and brings an interesting perspective with respect to recent publication on Pyrocystis noctiluca.
Thank you for this feedback. We've now added the following sentence to the discussion (line 274) to clearly communicate that many research questions about cell sinking *in situ* remain unanswered: "More broadly, it is important to note that the in situ vertical migration of phytoplankton is under significant influence by turbulence and ocean currents, and the importance of gravitational sinking of single cells to the movement of phytoplankton remains an open question."

(ii) The second discussion on cell size increase is a bit confusing since one claim from the paper is that density - not volume - is the main parameter driving sinking velocity changes. It thus seems counter-intuitive to relate the *small* changes in volume to arguments of cell surface area/resource intake.
Thank you for pointing this out. We've now removed any speculation about cell surface area to keep the discussion concise and well-justified.

(iii) The fourth paragraph rightfully clarifies that sinking may not be directly regulated but a byproduct of response to strvation. This statement is needed since some of the phrasing earlier in the paper suggests the opposite (i.e., 'phytoplankton increase sinking velocity in response to starvation'). However, the paragraph mixes a lot of ideas and is not easy to understand for non-specialists.
Thank you for pointing this out. We've now rewritten several parts of this paragraph, while also removing some sentences to maximize text clarity. The updated paragraph is copied below, and it now focuses explicitly on whether sinking is actively regulated and whether sinking influences cell fitness.
"It is possible that the changes in cell sinking that we observe following starvation do not necessarily reflect regulation of cell sinking, but rather byproducts of starvation-induced metabolic effects. The gravitational sinking velocity of most species studied here is under 1

m/day, which makes long distance (e.g., 50 m) vertical migration very slow and unlikely, especially for small cells. However, in a competitive environment, a fitness advantage might be gained by much more modest changes in depth. This is especially true for cells that inhabit depths close to the nutricline. In addition, gravitational sinking could be augmented by motility and cell aggregation, which were not studied here, but could also change in response to starvation. Our results also suggest that the increased cell sinking velocity following starvation could promote cell-to-cell encounters, possibly promoting cell aggregation and thus sinking."
